# Niosome-loaded antifungal drugs as an effective nanocarrier system: A mini review

**DOI:** 10.18502/cmm.4.4.384

**Published:** 2018-12

**Authors:** Mahmoud Osanloo, Sara Assadpour, Ahmad Mehravaran, Mahdi Abastabar, Javad Akhtari

**Affiliations:** 1 Department of Medical Nanotechnology, School of Advanced Technologies in Medicine, Fasa University of Medical Sciences, Fasa, Iran; 2 Student Research Committee, Faculty of Medicine, Molecular and Cell Biology Research Center, Mazandaran University of Medical Sciences, Sari, Iran; 3 Infectious Diseases and Tropical Medicine Research Center, Resistant Tuberculosis Institute, Zahedan University of Medical Sciences, Zahedan, Iran; 4 Department of Parasitology and Mycology, Faculty of Medicine, Zahedan University of Medical Sciences, Zahedan, Iran; 5 Department of Medical Mycology and Parasitology, Invasive Fungi Research Center, School of Medicine, Mazandaran University of Medical Sciences, Sari, Iran; 6 The Health of Plant and Livestock Products Research Center, Department of Medical Nanotechnology, Faculty of Medicine, Mazandaran University of Medical Sciences, Sari, Iran

**Keywords:** Antifungal, Drug Delivery, Liposome, Nanoparticle, Niosome

## Abstract

Skin is an important organ of the body due to offering an accessible and convenient site for drug administration. One of the disadvantages of transdermal drug delivery is the low penetration rate of drugs through the skin. Over the past decades, nanoparticles have been used as drug delivery systems to increase therapeutic effects or reduce toxicity. Encapsulation of drugs in nanoparticulate vesicles simplifies the transports of drugs into and across the skin.

Niosome nanoparticles are among these drug delivery systems, which have numerous applications in drug delivery and targeting. Niosomes are frequently used for loading drugs serving different purposes (e.g., anticancer, antiviral, and antibacterial agents). In recent years, there has been much research on the use of niosomal systems for the delivery of fungal drugs. A review of the literature investigating the advantages of niosomes in antifungal drug delivery can elucidate the efficiency and superiority of this nanocarrier over other nanocarriers.

## Introduction

Along with the development of nanotechnology, its applications in the medical and health sciences have increased dramatically. Recently, researchers widely use metallic nanoparticles (especially gold and silver) [1, 2], polymeric nanoparticles and fibers [3, 4], and lipid-based nanoformulations (e.g., nanoemulsions, solid lipid nanoparticles (SLNs), nanostructured lipid carriers (NLCs) and liposomes) [5-7] as drug carriers. Carrier is a special molecule or system used for the effective transportation of a loaded drug to preselected sites to serve targeted drug delivery. Carriers are engineered vectors, which retain drugs either on the cell surface or in a subcellular compartment via physical or chemical interaction, encapsulation, and spacer moiety [8]. 

Vesicles, especially lipidic ones, are used as carriers for drug delivery. Vesicles play a major role in modeling biological membranes; therefore, they are very useful in the transport and targeting of active agents through the membranes. Vesicular drug delivery system has some advantages over other targeted drug delivery systems. This system prolongs the existence of the drug in systemic circulation, and perhaps reduces the toxicity due to the delivery of drug directly to the site of infection. Both hydrophilic and lipophilic drugs can be incorporated into lipidic vesicles. 

Moreoever, lipidic vesicles delay the elimination of rapidly metabolizable drugs, thereby functioning as sustained release systems. Liposomes are simple microscopic vesicles with lipidic bilayer structures and an empty hole in the center entirely enclosed by a membrane. There are a number of components present in liposomes, such as phospholipid and cholesterol. The types of phospholipids include phosphoglycerides and sphingolipids [9, 10].

**Figure 1:**
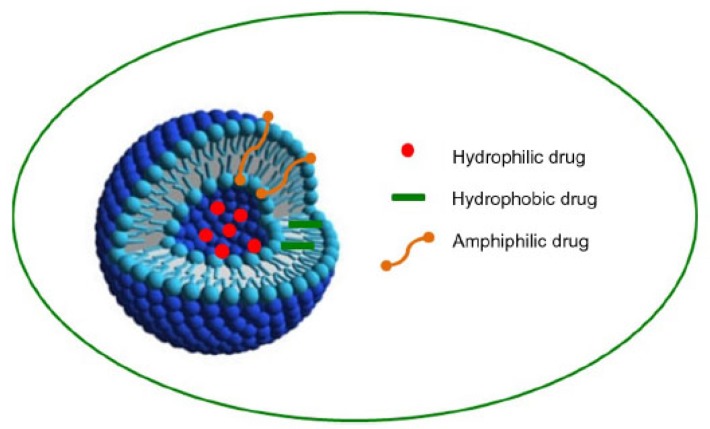
Structure of noisome and drug entrapment (hydrophobic drugs are generally localized in the outer shell or lipid layer, and hydrophilic core provides an ideal reservoir for hydrophilic drugs. Amphiphilic drugs are distributed in both parts.)

Recently, the use of self-assembled non-ionic surfactant based vesicles or niosomes has attracted wide attention in the field of topical, transdermal and targeted drug delivery. This results from the simple preparation, cost-effectiveness, chemical stability, high compatibility, biodegradability, and low toxicity (owing to their non-ionic nature) of these vesicles. In addition, enhanced solubility, permeability, and sustained release potentiality as local reservoir are the other benefits of niosomes [11-13]. 

The first niosome formulations were developed and patented by L’Oreal in 1975. Niosomes were first utilized in drug delivery for anticancer agents. Noisomes are capable of altering the pharmacokinetic profile, drug distribution in organ, and even drug metabolism. Niosomes have a core-shell structure and can carry both types of hydrophilic or hydrophobic compounds. Hydrophobic drugs are generally localized in the outer shell or lipid layer, while the hydrophilic core provides an ideal reservoir for hydrophilic drugs (Figure 1) [14, 15]. 

**Figure 2:**
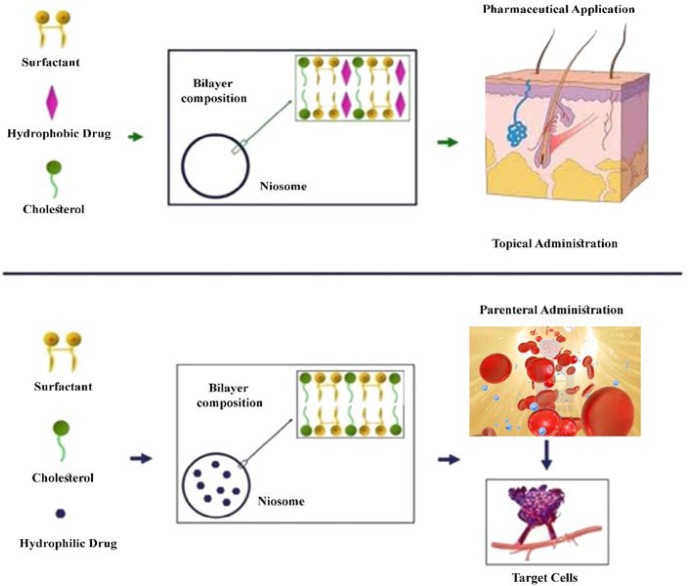
Formation of niosomes by non-ionic surfactant and cholesterol; entrapment of hydrophobic drug in vesicular membrane (up), entrapment of hydrophilic drug in aqueous part (down), topical administration to skin (up), and parenteral administration to target cells (down) (niosomes are similar to liposomes in having a bilayer.)

Niosomes contain two major components, including cholesterol and nonionic surfactants. Cholesterol provides rigidity and proper shape, while surfactants play a major role in the formation of niosomes. The non-ionic surfactants possess a hydrophilic head (non-polar) and a hydrophobic tail (Figure 2). The families of Spans (Span 20, 40, 60, 80, and 85), Tweens (Tween 20, 40, 60, and 80), and Brij (Brij 30, 35, 52, 58, 72, and 76) are commonly used as non-ionic surfactants in the preparation of niosomes. Niosomes are frequently used for loading different types of drugs. For instance, paclitaxel [16], acyclovir [17], and enoxacin/gentamicin [18, 19] are successfully loaded in niosomes as anticancer, antiviral, and antibacterial agents, respectively. 

Mortality rate attributed to invasive mycoses is on a growing trend. According to the statistics, in 1980, this group of diseases accounted for 828 deaths and was the 10^th^ most prominent cause of fatal infection in the US. However, based on the same dataset, the number of mycosis-related deaths increased to 2,370 in 1997, and this disease became the 7^th^ most prevalent terminal infectious disease [20, 21]. 

A majority of people suffer from superficial fungal infections in their lifetime. These types of infections are generally easy to cure; however, millions of people worldwide contract life-threatening invasive infections that are much harder to diagnose and treat [22]. Development of novel fungicides is a promising approach for controlling fungal diseases and overcoming resistant fungus [23]. Accordingly, over the past decades, efforts have been made around the world to develop highly efficient fungicides. With this background in mind, the present study was conducted to review the evidence on the use of niosomes as carriers of antifungal agents. The data are presented alphabetically according to the loaded drug.

***Amphotericin B***

Amphotericin B is a lipophilic antibiotic that is active against many filamentous fungi and yeasts*. *It is presumed that this medication alters the membrane permeability by binding with sterols in the fungal cell wall. Given the poor absorption of this drug by the gastrointestinal tract, it must be administered parenterally or incorporated into suitable carriers [24]. Sophorolipids have antimicrobial, antiadhesive, and antibiofilm properties. 

In a study explaining the preparation and characterization of niosomes with sophorolipids, this formulation was reported to have an entrapment efficiency of 63.20±3.86% for amphotericin B with a size of 80 nm. The mentioned study also involved the comparison of the efficacy of this formulation with that of a marketed drug, namely phosome (liposomal amphotericin B). Fewer hyphae were observed in the biofilm of *Candida albicans* treated with prepared sophorolipids-based niosomal formulation of amphotericin B, whereas more budding cells were found in the phosome-treated biofilm. Affordable production of noisome-based formulation and the suitability of this approach for the delivery of poorly soluble drugs, such as amphotericin B, against candidiasis are among the other advantages of this formulation [14].

***Clotrimazole***

Clotrimazole is a synthetic imidazole active against fungi (both dermatophytes and yeasts) and Gram-positive cocci (*Staphylococcus* and *Streptococcus* species), which can be used as an alternative to miconazole [24]. The proniosomal gel of clotrimazole is prepared by different non-ionic surfactants, such as Span 60, Span 40, Brij 72, and Tween 80, at a fixed drug dose (100 mg). Size of the prepared formulation is in a micron range, and their entrapment efficiency rates are reportedly 16% and 75% for Tween 80 and Span 60, respectively. Span 60 is shown to result in the best release by releasing 60% of the drug in 6 h. However, there is no report on the antifungal assay for the evaluation of the efficacy of this formulation [25].

***Diallyl disulfide and diallyl sulfide***

Diallyl disulfide (DADS) is a major organosulfur compound present in garlic with an antimitotic potential against neoplastic lesions. Consumption of DADS has been demonstrated to be associated with a low risk of gastrointestinal cancers in epidemiological studies [26]. Review of the literature resulted in the identification of two reports about the preparation of niosomes containing DADS and diallyl sulfide (DAS). In one of these studies, DADS loaded niosomes were prepared with a size of 140±30 nm. Hepatic and renal function tests, as well as histopathological studies, suggested that the formulation was safe at the investigated dose (12 mg/kg) in a model of candidiasis among BALB/c mice [27].

In the other report, the size and encapsulation efficiency of DADS loaded niosomes were 103-110 nm and 54-75%, respectively. Furthermore, niosomal DAS (administered 12 mg/kg body weight of Swiss albino mice) significantly reduced fungal load and mortality in treated animals, compared to the free form of DAS, and also was free of toxicity [28]. 

***Fluconazole***

Fluconazole is a triazole derivative with a broad spectrum of antifungal activity and rapid absorption. This drug readily passes across the blood-brain barrier into the cerebrospinal fluid. It has a plasma elimination half-life of about 30 h and is slowly excreted through the urine [24]. The literature contains few reports on the niosomal form of this medication. 

In the latest report, the ocular bioavailability of fluconazole was compared between two forms of niosomal gel and microemulsion in rabbits with fungal keratitis. The results of the mentioned study revealed that the bioavailability of both forms (i.e., niosomes [63.67-117.13 nm] and microemulsions [57.05-59.93 nm]) were significantly better than that of the solution form of the drug. However, niosomal gels were more sustainable and had a higher bioavailability (two folds) and an entrapment efficacy of 56.48-70.67% [13]. 

In a similar study, fluconazole-loaded niosomes were applied for the treatment of ocular infection in a topical form. Particle size of the formulations varied from 140 to 280 nm with an entrapment efficiency range of 40-84.35%. Bioassays showed that fluconazole had a significantly better antifungal activity in comparison with miconazole used as a positive standard [29]. There is evidence regarding the efficacy of liposomal and niosomal fluconazole preparations with the sizes of 348 and 326 nm and encapsulation efficiencies of 32% and 28%, respectively, against cutaneous candidiasis. The liposomal gel could produce 14.2-fold higher drug accumulation, compared with plain gel, while this variable was 3.3 folds more in the form of a niosomal gel [30].

Another study was focused on investigating the effect of surfactants on fluconazole-loaded niosomes. In the mentioned study, niosomes were prepared using film hydration method with different surfactants, namely Span and Brij. The results showed that the niosomes composed of Span 40, Span 60, and Brij 72 with the sizes of 378, 343, and 287 nm, respectively, had the highest stability with encapsulation efficiencies of > 41%. Release profiles after 12 h were 90% and up to 31% for the free and entrapped drugs, respectively [31].

***Griseofulvin***

Griseofulvin is a classical fungistatic antibiotic produced by *Penicillium griseofulvum*. This medication has a selective fungistatic activity against the dermatophytes causing ringworm (tinea) infections; nonetheless, it has no activity against pityriasis (tinea) versicolor or *Candida* species. It acts by disrupting the mitotic apparatus of fungal cells, thereby inhibiting protein synthesis [24]. Tinea infections are common dermatological conditions throughout the world. In a study comparing common griseofulvin gel with its niosomal and liposomal gel forms among 16 patients for 3 weeks, improvement was observed in all three groups. However, in the mentioned study, niosomal gel formulation produced the highest efficacy with minimal side effects [32].

***Itraconazole***

Itraconazole is a triazole derivative with a broad spectrum of antifungal activity. This drug is well absorbed and passes readily across the blood-brain barrier into the cerebrospinal fluid. The plasma half-life of this medication is about 30 h. It is metabolized in the liver and eliminated in the urine [24]. In a study examining itraconazole-loaded niosomes against *C. albicans, *the particle size and encapsulation efficiency of this formulation were reported as 124 nm and 60-90%, respectively. Furthermore, the zone of inhibition increased from ~ 10 mm in the marketed free drug to ~ 30 in niosomes [33].

***Ketoconazole***

Ketoconazole is a synthetic imidazole derivative with fungistatic activity against dermatophytes, yeasts, and other pathogenic fungi. This antifungal drug is widely used in the treatment of serious gastrointestinal and systemic mycoses, as well as in the management of superficial infections. It acts by inhibiting the synthesis of ergosterol, an essential component of the surface membrane of fungal cells [24]. Few reports are available on the evaluation of the antifungal activity of noisome-based formulation of Ketoconazole against *Aspergillus niger.* Size and encapsulation efficiency of niosomes had a range of 4860 to 7380±3.64 nm and 55.14-78.63%, respectively. Antifungal bioassays show the better efficacy of niosomal ketoconazole, compared to that of the conventional form [34]. 

***Miconazole***

Miconazole is a synthetic imidazole active against fungi (both dermatophytes and yeasts) and Gram-positive cocci (*Staphylococcus* and *Streptococcus* species) [24]. In a study, miconazole niosomes were prepared using thin film hydration method with varying ratios of cholesterol and surfactant. The prepared niosomes were in micron size range with an entrapment efficiency of 80-97%. The optimized formulation released 92% of the encapsulated drug in 24 h; however, the mentioned study contained no report on antifungal bioassay [35].

***Naftifine***

Naftifine is a primary topical allylamine that is available in a wide variety of formulations in many countries. This medication was the first topical antifungal agent in its class that held promise for the treatment of superficial fungal infections. In addition to the antifungal properties, this synthetic allylamine also shows significant antibacterial and anti-inflammatory properties, which may be particularly useful where superficial dermatoses are accompanied by superimposed bacterial infection and inflammation [36]. In a study targeted toward the preparation of the native alcohol-free niosomal gel, the formulation had a encapsulation efficiency of around 50% with a size of 90-150 nm. In the mentioned study, prepared niosomes were incorporated into a hydroxyethil-cellulose gel; however, their antifungal activities were not reported [37].

***Nystatin***

Nystatin is an antifungal polyene antibiotic derived from *Streptomyces noursei.* It is effective against the infections caused by a wide range of yeasts and yeast-like fungi [24]. Our review of the literature led to the retrieval of two reports about niosomal nystatin. In one of these studies, niosomal nystatin with a size rage of 164-395 nm was administered parenteally in neutropenic mice. The results of the mentioned research showed that the nisomal nystatin while having lower toxicity resulted in a significantly higher reduction in the fungal burden than free nystatin. 

For example, after 5 days of treatment, the reduction rates of fungal load in the liver were 92% and 26% in the mice receiving niosomal and free nystatin, respectively. Furthermore, these values were reported as 90% and 30% for the spleen, respectively [38]. In the other study, the researchers first prepared niosomal nystatin with a size range of 182-219 nm, and then prepared gel formulation using carbomers. The niosomal nystatin resulted in a prolonged drug release profile and a two-fold increase in drug deposition in the skin, compared to the conventional gel [39].

***Povidone iodine***

Povidone iodine (Betadine) is a complex of the potent bactericidal agent iodine and the carrier molecule povidone. The carrier complex slowly releases free iodine upon contacting with tissues. Povidone iodine is effective against Gram-positive and Gram-negative bacteria, fungi, and viruses [40]. In a study involving loading povidone-iodine in niosomes with a size of 338 nm as a carrier, a significant improvement was observed in permeability and retention of fungicidal activity against *C. albicans* [41].

## Conclusion

Over the past decades, nanoparticles have been used as drug delivery systems to increase therapeutic effects or reduce toxicity. Niosome nanoparticles are among these drug delivery systems, which have numerous applications in drug delivery and targeting. The use of niosomes can be very beneficial for the parenteral or dermal delivery of antifungal drugs. One of the disadvantages of transdermal drug delivery is its small penetration rate. 

Most of drugs are concentrated in the upper layer of the skin, namely stratum corneum. Encapsulation of drugs in nanocarriers, such as niosomes, simplifies the transport of drugs into and across the skin. Niosomes facilitate the enhancement of drug delivery through the skin. They may act as organic solvents for the solubilization of poorly soluble drugs. Beside, niosomes may serve as a local store for the sustained release of active compounds. 

Considering the penetration of surfactants into the stratum corneum, they may serve as penetration enhancers and facilitate dermal delivery [36]. The surfactants are biodegradable, biocompatible, and non-immunogenic. The most important reasons for administering niosomes for drug targeting via parenteral route include their delayed elimination from the circulation, potentiality for drug protection against biological environment, and exertion of restricting effects on target cells.

Based on the previous studies, the use of niosome as a drug carrier, especially for antifungal agents, results in better outcomes than the application of other carriers. Niosomes have important properties, including capability to encapsulate both hydrophilic and hydrophobic drugs and prolonged stability in circulation; moreover, they significantly enhance drug permeation through the skin. Due to the low cost of the raw materials (mostly surfactants), compared to that of the liposomes, they can be good candidates for the treatment of fungal diseases.
